# Evidence for selection on synonymous mutations affecting stability of mRNA secondary structure in mammals

**DOI:** 10.1186/gb-2005-6-9-r75

**Published:** 2005-08-16

**Authors:** JV Chamary, Laurence D Hurst

**Affiliations:** 1 Department of Biology and Biochemistry, University of Bath, Bath BA2 7AY, UK

## Abstract

**Background:**

In mammals, contrary to what is usually assumed, recent evidence suggests that synonymous mutations may not be selectively neutral. This position has proven contentious, not least because of the absence of a viable mechanism. Here we test whether synonymous mutations might be under selection owing to their effects on the thermodynamic stability of mRNA, mediated by changes in secondary structure.

**Results:**

We provide numerous lines of evidence that are all consistent with the above hypothesis. Most notably, by simulating evolution and reallocating the substitutions observed in the mouse lineage, we show that the location of synonymous mutations is non-random with respect to stability. Importantly, the preference for cytosine at 4-fold degenerate sites, diagnostic of selection, can be explained by its effect on mRNA stability. Likewise, by interchanging synonymous codons, we find naturally occurring mRNAs to be more stable than simulant transcripts. Housekeeping genes, whose proteins are under strong purifying selection, are also under the greatest pressure to maintain stability.

**Conclusion:**

Taken together, our results provide evidence that, in mammals, synonymous sites do not evolve neutrally, at least in part owing to selection on mRNA stability. This has implications for the application of synonymous divergence in estimating the mutation rate.

## Abstract 2

Simulating evolution and reallocating the substitutions observed in mouse genes revealed that in mammals synonymous sites do not evolve neutrally and synonymous mutations may be under selection because of their effects on the thermodynamic stability of mRNA.

## Background

At least in mammals, it is typically assumed that selection does not affect the fate of synonymous (silent) mutations, those nucleotide changes occurring within a gene that affect the coding sequence but not the protein [1,2]. This presumption is in no small part based on the understanding that effective population sizes (*N*_e_) in mammals are small. According to the nearly neutral theory [3], if *s *is the strength of selection against weakly deleterious mutations, then selection is expected to oppose their fixation when *s *> 1/2*N*_e _[4]. Consequently, when *s *is small, species with low *N*_e _are less likely to prevent the fixation of weakly deleterious mutations [5]. Indeed, for species with large effective population sizes, there is little doubt that selection is a strong enough force to determine the fate of synonymous mutations (for example, see [6]). Conversely, in mammals, analyses of codon usage have failed to detect clear signatures of selection (reviewed in [7]).

That synonymous mutations are effectively free of selection is important, not least because, if they really are neutral, their rate of evolution should be equal to the mutation rate. The rate of synonymous evolution could hence be used to provide a simple and convenient measure of the mutation rate [8,9]. More recently, however, the assumption of neutrality at synonymous sites has been called into question [10-16]. This view has proven contentious, not least because of the absence of a functional role for supposedly silent sites.

Here we examine one hypothesis, that synonymous mutations in mammals are under selection because they affect the thermodynamic stability of mRNA secondary structures [17,18], possibly to prolong cellular half-lives [19,20]. Unlike many non-coding RNAs [21-23], for which a stable secondary structure is selectively favored [24-28], the evolution of a stable structure for mRNA would be constrained by the need to encode a functional protein [17-19,29-31]. Consequently, were selection to operate on mRNA stability, synonymous mutations might be especially important (but see also [32,33]).

The hypothesis is supported by findings that synonymous mutations not only alter mRNA stem-loop structure [34,35], but also affect decay rates, and may lead to disease [35-37]. One possibility is that stem (base-paired) structures protect [38,39] against passive degradation by endoribonucleases [36,40,41]. Similarly, stable structures would be less likely to fall apart and thus expose vulnerable loop (single-stranded) regions to cleavage. Notably, analysis of computationally predicted mRNA stability across a wide taxonomic range revealed that real transcripts are more stable than comparable sequences in which synonymous codons were shuffled while the protein sequence remained unaltered [42,43].

Unfortunately, broad scale empirical analysis of mRNA stability is currently intractable because the structure of sequences much longer than tRNAs cannot be directly observed [20,44]. Consequently, mRNA folding is typically predicted computationally, by one of a variety of methods (see Materials and methods). Importantly, however, no *in silico *method can completely predict how cellular conditions might affect secondary structure [45]. For instance, proteins bound to mature transcripts [46] may have an effect, while chaperones are probably required to guide folding and/or prevent RNAs becoming kinetically trapped in unfavorable conformations [47,48]. Programs that attempt to incorporate the kinetics of the folding process that results from the directionality of transcription [49-51] are still under development [51]. Additionally, although a structure predicted *in silico *might be designated 'correct' because it forms *in vitro*, folding may be somewhat different *in vivo *[48,50].

The premise of this paper is not then to suppose that the prediction method and assumptions are flawless. Rather, we suppose that, if the method is telling us nothing about selection on mRNA stability, there is no reason why multiple independent tests should all point towards the same conclusion. In particular we ask: whether the nucleotides at synonymous sites are non-random with respect to stability; whether the excess of cytosine at synonymous sites in rodents [15] might be accounted for in terms of selection on mRNA stability; whether the location of substitutions in the mouse lineage are non-random with respect to stability; and whether genes under stronger purifying selection also have higher relative stability.

Although the hypothesis predicts that high mRNA stability should be favored, note that we do not expect stability to be extremely high, as ultra-stable structures would impose kinetic barriers that could hinder ribosome translocation [36,52]. While we presume that the transcripts of most genes will be relatively stable, in some cases mRNAs may actually need to be particularly unstable [43]. For example, selection might not act to promote stability because the mRNA is protein-bound and control of expression occurs at the translational level. Alternatively, some genes may only need to be transiently expressed, such as those encoding transcription factors [53,54]. As it is difficult to identify *a priori *which genes these might be, we cannot filter the dataset. This does, however, render our results conservative.

## Results

For 70 mouse mRNAs (Additional data file 1), we predict a single optimal putative secondary structure and its thermodynamic stability (ΔG, kcal/mol, the difference in free energy between the folded and unfolded states). Prior studies providing evidence of selection on mRNA structure have employed a randomization protocol that shuffles synonymous codons to generate numerous simulants [42,43,55,56]. Based on the idea that 'interesting' RNAs should be more stable than expected by chance [57], one can then ask whether the stability of a real (wild-type) transcript is, on average, greater than that of its simulants. Seffens and Digby [42], for example, did this for a range of taxa (from bacteria to human). To determine if there is a *prima facie *case to answer, we first performed an analysis similar to that done previously, but specifically restricted to mammalian sequences.

### Nucleotide content at synonymous sites is non-random with respect to mRNA stability

**Figure 1:**
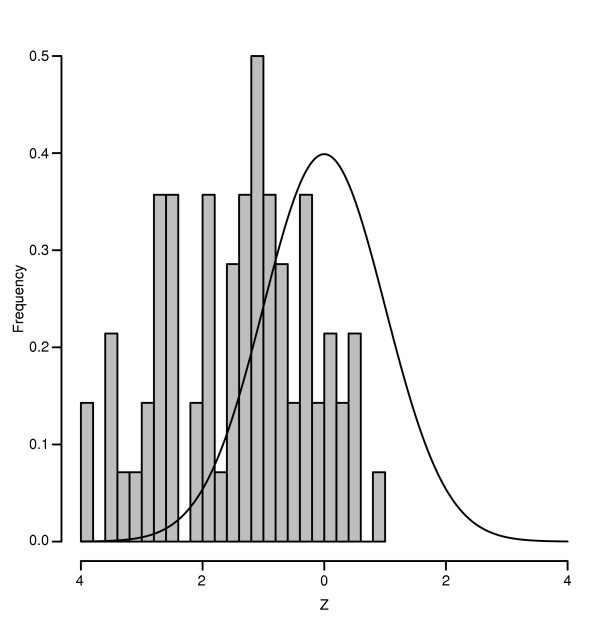
Stability of mRNA secondary structures for 'Sh.4-fold' simulants relative to real transcripts. Histogram of Z-scores for ΔG, the number of standard deviations the real mRNA is away from the mean stability of the simulants, following randomizations shuffling nucleotides at 4-fold degenerate sites (1,000 randomizations per gene, N = 70). The line shows the null normal distribution.

If selection acts on synonymous sites, by comparing a real mouse mRNA to simulants differing only at synonymous sites, we should find that, on average, the real transcript is more stable. For each gene we generated 1,000 random mRNAs identical in all regards to the real sequence, but with the bases at 4-fold degenerate (synonymous) sites in the coding sequence (CDS) randomly shuffled between the 4-fold degenerate positions. For each mRNA we determined Z(ΔG), the number of standard deviations the real mRNA is away from the mean stability of the simulants. Z(ΔG) is thus a measure of 'relative stability', the stability of a given mRNA relative to what one would expect by chance alone. Relative stability can also be considered as a measure of the strength of selection for stability, with a negative Z-score implying higher than expected stability. As Table 1 shows, real mRNAs are, on average, highly significantly more stable than 'Sh.4-fold' simulants (Figure 1; Additional data files 2, 3). Note, however, that on an individual basis, the effect (if any) is weak, with only 26 (37%) of genes having significantly high relative stability at the 5% level (Additional data file 4). Moreover, were we to apply Bonferonni correction for multiple testing on the by-gene *P*-values, no more than four genes would be significant at the 5% level. Inspection of the genes in our dataset (Additional data files 5, 6) did not reveal an obvious pattern that relates relative stability to their function.

In organisms from large effective populations, bias in codon usage is usually attributed to translational selection, favoring efficient (fast and/or accurate) protein synthesis as a consequence of skews in iso-acceptor tRNA abundance (reviewed in [7,58,59]). Whether this occurs in mammals, however, remains a contentious issue. While some have suggested that preferred sets of codons do exist to match the most abundant tRNAs [60], others maintain that codon usage does not reflect tRNAs skews [7,61] and that translational selection does not occur [62]. To be cautious, however, we also employed a protocol ('Sh.codon') that preserves the relative frequency of codons within a given set by shuffling codons within synonymous sets. This protocol gave very similar results to the previous ('Sh.4-fold') randomization (Table 1; Additional data files 2, 3).

### Cytosine preference at synonymous sites, diagnostic of selection, can be explained by selection on mRNA stability

While the above results suggest that the identity of the nucleotide at any given synonymous site is non-random, this need not reflect maintenance of mRNA stability. Selection could instead be acting on a thermodynamic property of DNA, such as bendability [63]. As more G:C pairings make helices more bendable and gene-dense regions are GC-rich (for example, see [64]), the putative selection on GC content we observe at the mRNA level might actually function to provide the transcriptional machinery with easier access to the most gene-dense regions of DNA. To address this issue, we asked about the strand-specific preference for cytosine at 4-fold degenerate sites observed in rodent exons [15].

Cytosine preference is indicated by two related features: a higher C content at 4-fold sites than in flanking introns (not observed for guanine) and an excess of C over G at 4-fold degenerate sites [15]. Correspondingly, we found C4 > G4 in 87% of our mouse genes and a mean skew in GC4 (G - C/G + C) of -0.1506 (*P *= 1e-11 for expected mean (μ) < 0 by one-sample t-test on GC4 skew). Importantly, the skew towards C is specific to exons and, therefore, cannot be accounted for by effects at the DNA level (for example, mutational biases such as transcription-coupled repair, or selection on transcription). Note also that the sign of the skew is the opposite of that derived from transcription-coupled repair, which yields a G excess [65,66]. Significantly, introducing synonymous changes that increase C|G dinucleotide content (where | is the codon boundary) extends mRNA half-life *in vitro *while increasing A|U enhances degradation [36]. If selection is acting on mRNA stability, then this could be explained by a high C content at third sites increasing the number of potential G:C base-pairs, which are stronger than A:U interactions (triple and double hydrogen bonds, respectively). Consistent with this, we find that genes with the highest relative stability also have a greater excess of C over G (Spearman rank correlation coefficient (ρ) = 0.27, *P *= 0.0225 for GC4 skew versus Z(ΔG^Sh.4-fold^); Additional data file 7).

To further examine the possibility that the C preference is explained by selection on RNA structure, we also asked whether replacing C residues with G decreases stability. We found that real mRNAs are more stable than modified transcripts in which, at 4-fold sites, we swapped all Cs for Gs and vice versa (Table 1; Additional data files 2, 3). 'Swap G4C4' mRNAs, however, possess a higher percentage of base-pairs than real transcripts (62.11 ± 0.33% and 60.96 ± 0.28% in CDS, respectively, *P *= 0.0003 by paired t-test; 60.84 ± 0.26% and 61.61 ± 0.26% in mRNA, *P *= 0.0007). That 'Swap G4C4' mRNAs have more base-pairs but lower stability can be explained by the existence of G:U base-pairs within stems, as G:Us are weaker than Watson-Crick interactions (A:U and G:C). An increased G content increases the amount of G:U pairs (real 10.50 ± 0.26% and 'Swap G4C4' 11.64 ± 0.21% in mRNA, *P *= 3e-07) and thus the proportion of base-paired mRNA, but their stems are less stable (there is no difference in the proportion of A:U pairs: real 36.60 ± 0.70%, 'Swap G4C4' 36.39 ± 0.71%, *P *= 0.2449). These results further underline the importance of nucleotide content for mRNA rather than DNA stability, not least because the location of bases that can potentially form Watson-Crick base-pairs in DNA is preserved in the modified transcripts.

### Biased amino acid content and RNA stability may together drive C preference at third sites

The results above suggest that, given the nucleotide content at non-synonymous sites, C enrichment at synonymous sites is adaptive in regards to mRNA stability. Is there something about non-synonymous sites that causes C in particular to be enriched at synonymous sites? Fitch [17] proposed that, if genetic code degeneracy is exploited to optimize base-pairing in mRNA, third sites within codons (usually synonymous) should be preferentially paired with first and second sites (few and no synonymous sites, respectively). This would also provide a buffer for mRNA structure against non-synonymous substitutions via compensatory changes. Cytosine preference at third sites might, therefore, be driven by selection on amino acid content and mRNA stability [19].

In stems, we expect that, to permit base-pairing, a high G content at first and second sites should be matched by a high C content at third sites (and vice versa), that is, selection on non-synonymous sites would, at least in part, dictate nucleotide content at synonymous sites. At base-paired sites in mRNAs, there is a strong negative correlation in GC skew between first/second sites and third sites (for example, Pearson correlation coefficient (*R*) = -0.65, *P *= 1e-09 for GC12 skew versus GC3 skew) that is not observed at unpaired sites (*R *= 0.70, *P *= 1e-11; Additional data file 7; note that a positive correlation is expected from isochore structure [67]).

Given the potential inaccuracies of minimum free energy prediction methods (see Materials and methods), we also asked whether the above relationship is robust to the exclusion of sites at which one is less confident that base-pairing occurs (either with a particular site in the optimal structure or with any other site). GC skew is then only calculated for those sites where the probability of pairing is greater than some minimum threshold. We found that the significant negative correlation in GC skew between first/second and third sites is strikingly insensitive to different threshold values (Additional data file 8).

Jia *et al*. [68] recently observed that α-helices and β-sheets of protein secondary structures are preferentially 'coded' by mRNA stems. Using data on the amino acid preferences for protein conformations [69], we found G to be more abundant than C at first and second sites in both α-helices and β-sheets (GC12 skews of 0.001 and 0.0420, respectively). Similarly, there is a bias towards G in these regions within the proteins from our dataset (α-helix GC12 skew of 0.0608 ± 0.0143, *P *= 8e-05 for μ = 0 by one-sample t-test; β-sheet skew of 0.0879 ± 0.0312, *P *= 0.0102). The C preference at third sites may, therefore, reflect selection to maintain stable stems in these regions enriched for G at largely non-synonymous sites.

### The location of observed synonymous substitutions is non-random with respect to mRNA stability

While randomization protocols that shuffle or swap nucleotides provide insights into how putative selection for mRNA stability and nucleotide content interact, these processes do not occur in nature. The most direct evidence that we can consider is to examine the locations of observed synonymous mutations. Reallocating point mutations is a more realistic form of analysis as it mimics the process of selection following mutation (nucleotide substitutions that are not the result of single point mutations are very rare in mammals, for example, see [70-73]). This minimizes potential biases. For example, randomization protocols that shuffle nucleotides or codons (for example, see [42]) might be problematic [74] as they generate a large number of variants in which there will be a profound effect on dinucleotide relative abundances [75-77]. Simulating the process of evolution, however, only introduces 7 to 8 synonymous changes per 100 sites, hence only about 1 to 2 per 100 nucleotides in the coding sequence. This will have negligible impact on dinucleotide distribution.

Parenthetically, as recent evidence suggests that dinucleotide content in rodent exons is the result of selection [15] and not of biased mutation and/or repair [56,75], the desirability of controlling for dinucleotide distribution is highly questionable. Put differently, if a real mRNA is on average more stable than expected when compared to simulants in which the observed point substitutions have been reallocated, biased dinucleotide distribution is more likely to be a consequence of selection for favorable base-stacking interactions rather than mutational/repair biases.

If certain mutations really were under selection because they diminished mRNA stability, relocating those substitutions actually seen to random locations ('Re-sub.') should lower stability. We used parsimony to determine the substitutions that have arisen in the mouse lineage, inferring the CDS of the rat-mouse common ancestor using hamster as the outgroup to maximize reliability and the number of informative sites (Additional data file 9). We reverted all synonymous changes back to the ancestral state and then simulated mutation by randomly reallocating substitutions at synonymous sites, maintaining the number of observed changes and the encoded protein.

Note that the application of parsimony, while a common practice in the mouse-rat comparison (for example, see [78,79]), can sometimes provide biased ancestral state reconstructions (for example, see [80]). We therefore also reconstructed rat-mouse common ancestor sequences using a maximum likelihood approach. At only 3 of 86,334 reconstructed sites did the parsimony and maximum likelihood methods disagree (excluding sites differing in all three species, see Materials and methods). All three discrepancies occurred in the same gene (*Gadd45a*). Exclusion of this one gene makes no difference to our results (Additional data file 2).

As nucleotide content is influenced by genomic location (isochores; for example, see [67]), the re-introduced nucleotides were selected at random, but in proportion to base composition at third sites in the appropriate mouse gene. This also further minimizes the negligible effect on dinucleotide distributions. From this randomization ('Re-sub.N3') we again find that real mRNAs are, on average, more stable than expected by chance (Table 1; Additional data files 2, 3). Ignoring the effect of isochores and changing the profile of permitted substitutions does not qualitatively alter this result. For example, allowing all mutations to occur with equal likelihood ('Re-sub.*K*') also shows that the locations of observed substitutions have had minimal impact on stability (Table 1; Additional data files 2, 3). Simulants and real transcripts possess a similar amount of base-pairs (*P *> 0.15 by one-sample t-tests on Z(%base-pairs), μ = 0; Table 1).

### Signals of selection or methodological artifact?

While the above results indicate that the location at which certain synonymous mutations are observed is in part determined by constraints on mRNA stability, could the above results be artifacts of an inaccurate methodology? We have attempted to minimize such problems by considering those sequences in which *a priori *we expect the method to be more accurate and by considering only those sites that have a high probability of being base-paired. We can, however, consider additional tests. If selection for mRNA stability occurs, we also expect that substitution rates should be related to predicted stem-loop structure and that genes known to be under strong purifying selection should possess mRNAs with high relative stability. We examine these two predictions in turn.

### Genes with a high proportion of base-pairs may have fast-evolving stems: evidence for compensatory substitutions?

Testing the first prediction, that evolutionary rates should be linked to mRNA secondary structure, is not straightforward, even if structure prediction were perfect. Although one expects that the majority of compensatory changes will occur to restore substructures, the thermodynamic hypothesis posits that some will also act to restore the overall stability of the molecule. Even if a precise secondary structure were conserved, the difficulty lies in the fact that a given substitution can only be assigned to having occurred within a stem or loop before or after it potentially affects base-pairing, for example, a transversion at a base-paired site in the ancestral mRNA will create a bulge/loop. Consequently, the only substitutions that can be observed within the same (conserved) structure of the descendant sequence are those that arise within loops with little stem-forming potential or within stems in which a compensatory substitution has restored complementary base-pairing. With this caveat in mind, we examined observed substitutions with respect to the predicted secondary structure in mouse.

We first asked whether substitution rates correlate with the percentage of sequence involved in base-pairing interactions. We found that both the number of synonymous substitutions per synonymous site (Ks) and the non-synonymous substitution rate (Ka) for the whole CDS are higher in genes with more base-pairs (*K*_a _ρ = 0.31, *P *= 0.0091, N = 70; *K*_s _ρ = 0.31, *P *= 0.0101, N = 69), although the result for non-synonymous mutations is sensitive to restricting analysis to the subset of small mRNAs (Additional data file 10). These effects seem to be a consequence of substitutional processes within stems. While there is a positive correlation between %base-pairs and rates within putative stems (*K*_a _ρ = 0.31, *P *= 0.0090, N = 69; *K*_s _ρ = 0.37, *P *= 0.0020, N = 68), no such relationship exists in loops (*K*_a _ρ = -0.03, *P *= 0.7941, N = 69; *K*_s _ρ = -0.03, *P *= 0.8264, N = 69; Additional data file 10).

Note that these latter correlations do not mean that stems evolve faster *per se *(one would predict the opposite), only that they may evolve faster when a lot of the sequence is base-paired. Indeed, consistent with stems being under purifying selection to maintain secondary structure, while non-synonymous rates are the same between codons in putative stems and those in loops (*P *= 0.6233, N = 69 by paired t-test, stem = 0.0110 ± 0.0018, loop = 0.0095 ± 0.0012), synonymous sites in loops evolve 37% faster than those in stems (*P *= 0.0045, N = 68, stem = 0.0833 ± 0.0071, loop = 0.0608 ± 0.0034; Additional data file 10).

Why might a high proportion of base-pairing be associated with rapid substitution rates within stems? One possibility is that an abundance of base-pairs ensures that no single mutation can grossly destabilize an mRNA. While one might then predict a negative correlation between %base-pairs and Z(ΔG) (that is, changes to mRNAs with little secondary structure will have a large impact on stability), this may not be observed because when substitutions are randomly reallocated the majority will not fall within stems. Alternatively, the relationship between %base-pairs and substitution rates within stems may indicate a high rate of compensatory changes restoring stem structures. Consider a mutation that arises within a stem that destabilizes the mRNA secondary structure. If selection maintains transcript stability, the substitution will only be tolerated if it is adaptive at the protein level or has such a negligible impact on stability as to be effectively neutral. In the latter case, further changes could accumulate that in combination might significantly alter structure. Under both scenarios, subsequent compensatory mutations restoring stability would thus be under positive selection. The effect of one mutation arising within a stem that has the knock-on effect of increasing substitution rates within stems would be most pronounced in genes with a high proportion of base-pairing. Consequently, compensations would be most favored when there is high pressure to maintain stability. Indeed, we find that in those genes under the strongest selective pressure for high stability, putative stems are fast-evolving (ρ = -0.37, *P *= 0.0020 for Z(ΔG^Re-sub.N3^) versus *K*_s_, N = 68).

### Housekeeping genes have high relative stability

To test the second prediction, it is necessary to define *a priori *a set of genes likely to be under stronger purifying selection. Prior evidence indicates that genes expressed in most tissues, housekeeping genes, may be good candidates for two reasons. First, housekeeping proteins evolve slower than tissue-specific ones [73,81-83]. Second, experimental assays of half-life have demonstrated that mRNAs of housekeeping genes degrade relatively slowly [53,54].

Here we identify housekeeping genes by calculating the breadth of expression, the proportion of tissues in which a given gene is expressed. We call a gene 'expressed' in a particular tissue if the average hybridization intensity on microarrays ('average difference' (AD)) for the transcript is greater than 100 or 200 (approximately 2 or 4 copies per cell, respectively, [84]). Housekeeping genes are those expressed in a large proportion of tissues. As described previously (for example, see [73]), we found that protein evolution is slowest in housekeeping genes (%tissues versus *K*_a_: ρ = -0.39, *P *= 0.0008 for AD > 200; ρ = -0.32, *P *= 0.0065 for AD > 100).

Significantly, consistent with the prediction, we found that genes subject to strong purifying selection (housekeeping genes) also have the highest relative stability, with the inferred intensity of selection on mRNA stability being correlated with breadth of expression in the expected direction (ρ = -0.25, *P *= 0.0335 for %tissues versus Z(ΔG^Re-sub.N3^) at AD > 200). Using a less conservative cut-off to define a gene as expressed (AD > 100) increases the strength and significance of the correlation (ρ = -0.29, *P *= 0.0159). The relationship becomes more pronounced after controlling for sequence length (partial ρ = -0.25, *P *= 0.0179 for AD > 200; partial ρ = -0.30, *P *= 0.0069 for AD > 100; significance determined by 10,000 randomizations). Expression breadth is not associated with the proportion of the sequence that is base-paired (ρ = -0.01, *P *> 0.9 for %tissues versus %base-pairs in CDS), nor does the amount of base-pairing predict relative stability (*R *= -0.14, *P *= 0.2630 for %base-pairs versus Z(ΔG^Re-sub.N3^)). As suggested from the 'Swap G4C4' modification protocol, this supports the importance of overall stability over the amount of secondary structure.

## Discussion

We have provided numerous lines of evidence that support the hypothesis that selection on synonymous mutations can be mediated by effects on mRNA stability in mammals. Importantly, the signature of selection in rodents, the C preference at 4-fold degenerate sites [15], can potentially be explained by selection on synonymous mutations affecting mRNA stability. That it should be C in particular (rather than A, G or T), is further explained by skews in nucleotide usage at largely non-synonymous sites: G enrichment at the first and second sites in codons is matched by C enrichment at third sites, so as to ensure, we argue, strong G:C pairs in the mRNA. Moreover, through a randomization that simulates evolution in the mouse lineage, we show that, had the observed substitutions occurred elsewhere within a sequence, they would have had a greater impact on mRNA stability. Additionally, not only do housekeeping genes have unusually low rates of protein evolution, their mRNAs have unusually high relative stability, both features being consistent with stronger selection on this class of genes. Although the structure prediction tool is by no means perfect, it is not obvious how it could be biased in such a way as to cause all our results to point towards the same conclusion.

Synonymous mutations can also be under selection for other functions. Can we be confident that these effects are independent? Recent evidence also suggests that a preference for exonic splicing enhancers (ESEs) affects codon choice [85,86] and that ESEs are under selection [87]. It is likely, however, that the results presented here and selection on ESEs are independent, as ESE hexamers are rich in G compared with C (24% and 14%, respectively, see [86] for dataset), while mRNA stability appears to explain high C content. Moreover, ESEs define relatively little sequence, being short and predominantly located within 20 nucleotides of splice junctions [87].

### Experimental predictions for selection on mRNA stability

One might suppose that *in silico *simulations could explain variation in decay rates between genes. Z(ΔG) is not a measure of absolute stability, however, but rather of stability relative to what might have been observed given the underlying parameters of a gene, such as length and coding capacity. Only if all such parameters were equal between genes would one expect relative stability to predict decay rate. However, all else is not equal; for example, we find that Z(ΔG) and nucleotide content covary. Therefore, looking for a correlation between Z(ΔG) and half-life [56] is a weak test because an absence of a relationship would not be strong evidence against the hypothesis unless other variables could be controlled. Indeed, results are ambiguous. Mammalian housekeeping genes have longer half-lives [53,54] and we find that they also have high relative stability. In contrast, Katz and Burge [56] found no correlation between decay rate and local Z(ΔG) in yeast. The interpretation of the yeast result is made even less clear due to uncertainty over when mRNAs should be folded globally. The issue might be easier to resolve once high-quality non-human sequence from primates becomes available, as one could then compare available large-scale surveys of human mRNA decay rates (for example, see [54]) with relative stability. As hominid *N*_e _is around an order of magnitude lower than in murids [88], however, it is also conceivable that selection may not be strong enough to act on mRNA stability.

On the other hand, simulations should predict relative decay rates of mutant versions of a given gene. In at least one case, the dopamine receptor D2 gene, it has been demonstrated that only single nucleotide polymorphisms that induce a conspicuous change in structure predicted *in silico *affect mRNA half-life *in vitro *[35]. A much larger sample set is required to determine whether this is more generally true. We predict that, for those genes with the highest relative stability, the real mRNA should have a longer half-life than the majority of mutants in which one has randomly reallocated synonymous mutations.

### Implications for understanding codon usage and mutation rates

That selection maintains mRNA stability contradicts the accepted wisdom that synonymous mutations evolve neutrally [1,2], not only because changes do not alter protein sequence, but also because mammalian effective population sizes (*N*_e_) are thought to be too small to permit selection on mutations of small effect on fitness [6]. Moreover, nucleotide content at silent sites in mammals is best predicted by genomic location (isochores; for example, see [67]). Our observations, however, nonetheless tally with recent evidence that selection acts on synonymous mutations [10-16].

Selection favoring accurate or fast protein synthesis, the classically cited functional role for biased usage of synonymous codons, is not well supported in mammals [7,61,62]. Translational selection predicts that highly expressed genes should exhibit the greatest bias in codon usage [7], but the effect is only weak [13,60,89] and a bias is also observed in lowly expressed genes [89]. On the other hand, selection for mRNA stability need not correlate with expression level (indeed, we find no relationship between Z(ΔG) and mean or peak expression level; *P *> 0.1 in all cases).

When translational selection is known to occur, it can be at odds with selection for mRNA secondary structure (fly, [20]) and stability (yeast, [90]), leading to a trade-off between the two forces [20,90]. Given the difficulties involved in detecting codon usage bias in mammals [7] and our results above, we infer that selection on mRNA stability must be strong relative to translational selection (if the latter occurs at all). This has two repercussions. First, selection for mRNA stability could, in principle, weaken any signal of a preferred set of codons for translational efficiency. Second, in terms of detecting selection at synonymous sites in mammals, asking whether a given amino acid always prefers a certain codon is not necessarily asking the right question. Indeed, it is quite possible that there exist no preferred codon within a gene while at the same time synonymous mutations are under selection. More generally, a complex set of trade-offs between different forms of selection and mutational biases may render interpretation of patterns of codon usage very difficult.

The evidence for selection on synonymous mutations also has implications for our understanding of both the mutation rate and the mutational load. The substitution rate at synonymous sites in exons is often used as a measure of the mutation rate [8,9]; however, this assumes neutral evolution of synonymous mutations [1,2]. By providing a parsimonious mechanism by which selection could act on synonymous sites, we can ignore the objection that prior evidence is indirect. Nevertheless, it is presently unclear to what degree synonymous mutations are favored or opposed by selection due to their effects on mRNA stability. Without being able to quantify the latter, as well as the net effect of other biases (for example, splice-associated), it will not be possible to directly estimate the extent to which use of the synonymous substitution rate leads to underestimates of the mutation rate and the mutational load.

## Conclusion

Recent evidence has suggested that, despite assumptions to the contrary, synonymous mutations in mammalian exons can be under selection. Here we have provided several independent lines of evidence to support the notion that this effect may in part be mediated by selection for mRNA stability. Notably, the preference for cytosine at synonymous sites can be accounted for by such a process. Importantly, the observed substitutions appear to be present at particular sites so as to avoid affecting mRNA stability. Our results have implications for the manner in which codon usage bias should be analyzed to detect selection and for attempts to estimate the mutation rate.

## Materials and methods

### Orthologous rodent genes

We identified gene families from HOVERGEN (Release 44) [91,92] with complete CDSs for *Mus musculus*, *Rattus norvegicus *and hamster. Orthology was defined as the topology (((mouse, rat), hamster), non-rodent outgroup) within the phylogenetic tree for a given gene, without intervening non-rodent branches between the rodents. Seventy well-described genes matched these criteria and had a <5% size difference between the longest and shortest CDS. Non-redundancy and orthology were supported by syntenic comparisons [93]. Unless otherwise stated, N = 70 for all statistical tests.

### Mouse mRNA sequences

Accession numbers from HOVERGEN were used to extract mRNAs from the EnsEMBL genome assembly (Build 30) [94]. When alternative transcripts existed, we used the rat and hamster sequences to identify the desired exons. The untranslated region (UTR) database (Release 15) [95,96] was used for six genes because the UTRs in the EnsEMBL files were unreliably annotated. If present, poly(A) tails were removed as they are coated with binding proteins and so are unlikely to be involved in base-pairing [97].

### Coding sequence alignments

Each CDS was extracted using GBPARSE [98] and translated. We aligned amino acid sequences as previously described [15] then reconstructed the three-way nucleotide alignment using AA2NUC (available from L.D.H.).

### Reconstruction of rat-mouse common ancestor sequence

Parsimony and maximum likelihood were used to reconstruct ancestral sequence. At 0.3% of sites in the rodent alignment, the rat-mouse ancestral state could not be determined (for example, a different base was present in each species). In these cases, we used the mouse sequence to be conservative for the number of substitutions that have occurred in the mouse lineage. Ancestral states derived from maximum likelihood were determined using codeml in the PAML package [99,100].

### RNA secondary structure prediction

There are two main computational approaches to predicting RNA secondary structure. The first is a thermodynamic method, which assumes that a given sequence will fold into the structure with the minimum free energy [101]. The second approach compares multiple orthologous sequences to identify patterns of co-evolution between sites that could be indicative of compensatory mutations [102] to maintain complementary base-pairing within stems [103-108].

In the context of our analysis, the choice is highly constrained and comparative methods may not be applicable to the hypothesis we test. Comparative methods require all input sequences to be of high quality and for the alignment to be accurate. Here we are particularly interested in knowing where substitutions have occurred in a given mammalian lineage and, therefore, need sequence from three species, with mouse-rat-hamster being the obvious choice. Currently, however, rat genomic sequence is not of sufficiently high quality and annotation of UTRs is unreliable. UTRs from hamster are largely unavailable.

Although a moot point under the above circumstances, it may also be undesirable to apply a comparative method in the current context, not least because the logic would be circular: the method requires us to assume that selection is strong enough to maintain secondary structure, while at the same time we are testing for selection. More importantly, based on the evolution of non-coding RNAs, comparative methods are geared towards detecting secondary structure that has been conserved despite sequence divergence [49], that is, well-conserved substructures exist which tend to have specific functions (for example, the anti-codon within a tRNA must always be within a loop). For mRNA, however, a more realistic model is that selection favors the stability of the mRNA conformation as a whole [17,18]. Highly conserved substructures are not expected *a priori *[109], in part because such conservation may not always be possible, as protein-coding function should outweigh any RNA structure considerations. Essentially, the model assumes that the mRNA will adopt the optimal structure given the available sequence.

Structure and stability were predicted using RNAfold from the Vienna package (Version 1.4) [110,111] under default settings (folding at 37°C, tolerating non-Watson-Crick G:U pairs). Thermodynamic parameters were derived experimentally [112]. RNAfold implements an algorithm that, for a given RNA, finds the conformation with the minimum free energy by maximizing favorable base-pairing interactions [101].

### Global versus local mRNA stability

A second methodological issue concerns whether selection might act on stability at the local or global scale. There are two critical issues when choosing which to assess. First, if opposite ends of a molecule are able to pair with one another, RNAs may adopt a conformation closer to a global optimal structure. In eukaryotes, unlike bacteria (where transcription and translation are simultaneous and co-localized), long-range interactions between opposite ends of mRNA molecules can occur [113-116]. This suggests that global [20] rather than local stability is more important to analysis of mammalian sequence.

Second, one must also ask whether the genes contain introns. Generation of a globally stable structure would require the action of spliceosome-associated helicases (for example, [117-119]) to maximize the amount of available sequence. Indeed, it is significant that intronless genes in yeast are less biased for structure than those with introns [56]. All genes in our dataset contain introns, further suggesting global stability to be the more relevant measure. Nonetheless, our assumption of global maximum stability, while an appropriate functional hypothesis, may at best only be a good approximation, as in some cases (for example, short transcripts) there may not be enough time for an mRNA to discover the most optimal structure.

### Controling for sequence length

While minimum free energy predictions often agree with laboratory-based methods (for example, stem-loops are avoided at the AUG initiation codon, [120-123]), they are less reliable for long sequences (for example, [112]). The mean length of transcripts in our dataset is 2,101.41 ± 139.84 nucleotides (nt). Consequently, where relevant, we endeavored to control for length effects. In most cases, we carried out the same analyses for mRNAs shorter than 2,000 nt (N = 36, mean mRNA length of 1219.38 ± 77.32 nt), this being the cut-off defining two halves of the dataset. Through Mantell simulations, we found that, when testing for selection on stability, in no instance is the *P*-value for the smaller dataset both not significant and higher than that expected if one were to randomly sample half the dataset, where the full data set analysis suggested significance (Additional data file 3). Consequently we conclude that the results are not obviously biased by the inclusion of long sequence.

### Protein function and secondary structure prediction

The attributes of mouse gene products were obtained from the Gene Ontology database (June 2004) [124].

Amino acid sequence was designated as occurring in α-helix, β-sheet (strand) and coil regions using PSIPRED (Version 2.3) [125,126] under default parameters (masking low complexity regions).

### Rates of evolution

The number of non-synonymous substitutions per non-synonymous site (*K*_a_) and the synonymous (*K*_s_) distance were estimated with the Li method [127] using the Kimura 2-parameter model. We excluded one fast-evolving gene (*K*_a _= 0.5; *K*_s _= 0.17) in our analyses of evolutionary rates, although inclusion of the outlier gave similar results.

### Coding sequence randomization protocols and statistical significance

Simulant mRNAs are identical to their real counterparts in their 5' and 3' untranslated regions and the encoded protein.

On a single-gene basis, the significance of whether its mRNA is more stable than expected by chance is given by:



R is the number of artificial mRNAs that are more stable than the real transcript, N is the number (1,000) of randomizations (see Box 1 in [128]).

The Z-score for stability is given by:



The Z-scores derived from all randomization protocols are normally distributed.

### Expression

Cellular mRNA levels from normalized microarray data on Affymetrix chips were obtained from SymAtlas [129]. We identified the expression profile for each gene by BLASTing mRNA sequences against the probes for the GNF1M chip [130], which has measurements from 61 non-redundant tissue types (the five 'embryo' tissues were ignored). We used the 45-tissue dataset [84] from the U74A chip for six genes where the suggested BLAST hit from GNF1M were not syntenically feasible. For each tissue we took the mean level across replicate hybridizations. Breadth was set to 0 if AD < 50 in all tissues.

### Mantell simulations

To determine whether the incorporation of long genes substantially biased our results, for each modification/randomization protocol, we considered the effect of removing the half of the dataset containing the longest genes. Given that this subset of small mRNAs is by necessity half the size of the full dataset, it is inevitable that *P*-values will be increased. The issue is whether they have increased more than would be expected had we randomly sampled half the dataset. To this end, we randomly extracted 36 genes and re-calculated the significance from t-tests. This was repeated 10,000 times per modification/randomization protocol, yielding the underlying distribution in *P*-values that would be expected were sequence length unimportant. The observed *P*-value (for the shortest genes) was then compared to this expected distribution (see Additional data file 3).

## Additional data files

Additional data are available with the online version of this paper. Additional data file 1 contains sequences for all 70 mouse mRNAs in FASTA format. Additional data file 2 is equivalent to Table 1, but excludes the one gene (*Gadd45a*/HBG000516) where the rat-mouse common ancestor sequence differed slightly using the parsimony and maximum likelihood reconstructions. Additional data file 3 is equivalent to Table 1, but only considers mRNAs shorter than 2,000 nucleotides. Additional data file 4 provides the stabilities, relative stabilities and significance values for each modification/randomization on a by-gene basis. Additional data file 5 contains various sequence identifiers (for example, accession numbers) for each mouse gene. Additional data file 6 features gene ontology information, including a description of the function of each mouse gene product. Additional data file 7 contains various correlations for short genes, including GC4 skew versus Z(ΔG^Sh.4-fold^), GC12 skew versus GC3 skew (separately for base-paired and unpaired sites) and Z(ΔG^Re-sub.N3^) versus *K*_s _at base-paired sites. Additional data file 8 is a table of correlations between GC skew at first/second sites versus skew at third sites, provided for a series of thresholds where the sites analyzed must have a minimum probability of base-pairing. Additional data file 9 is a FASTA file containing three-way alignments of coding sequences from hamster, rat and mouse orthologous genes. Additional data file 10 is a table of correlations for short genes, between the proportion of base-paired sites and non-synonymous or synonymous substitution rates within the coding sequence, base-paired sites and unpaired sites.

## Supplementary Material

Additional data file 1 Sequences for all 70 mouse mRNAs in FASTA format. Click here for file

Additional data file 2 A table of the stability of mRNA secondary structures excluding the *Gadd45a *gene, where the rat-mouse common ancestor sequence differed slightly using the parsimony and maximum likelihood reconstructions. Click here for file

Additional data file 3 A table of the stability of mRNA secondary structures for short genes (only considers mRNAs shorter than 2,000 nucleotides). Click here for file

Additional data file 4 Stability and relative stability values for individual genes. Click here for file

Additional data file 5 Sequence identifiers for each mouse gene. Click here for file

Additional data file 6 Ontology information for each mouse gene. Click here for file

Additional data file 7 Miscellaneous correlations for short genes, including GC4 skew versus Z(ΔG^Sh.4-fold^), GC12 skew versus GC3 skew (separately for base-paired and unpaired sites) and Z(ΔG^Re-sub.N3^) versus *K*_s _at base-paired sites. Click here for file

Additional data file 8 A table of correlations between GC skew at first/second sites versus skew at third sites, provided for a series of thresholds where the sites analyzed must have a minimum probability of base-pairing. Click here for file

Additional data file 9 A FASTA file containing three-way alignments of coding sequences from hamster, rat and mouse orthologous genes. Click here for file

Additional data file 10 A table of correlations for short genes, between the proportion of base-paired sites and non-synonymous or synonymous substitution rates within the coding sequence, base-paired sites and unpaired sites. Click here for file

## Figures and Tables

**Table 1:** Stability of mRNA secondary structures

	Protocol	Mean ΔG	*P*	Mean Z(ΔG)	Mean %pairs
Real (mouse)		-737.98 ± 55.52			60.96 ± 0.28
Modification	Swap G4C4	-734.10 ± 55.08	0.0169		62.11 ± 0.33
Randomization	Sh.4-fold	-725.76 ± 54.71	9e-15	-1.41 ± 0.14	60.77 ± 0.23
	Sh.codon	-728.49 ± 55.01	6e-10	-1.04 ± 0.14	60.61 ± 0.23
	Re-sub.*K*	-733.28 ± 55.15	4e-05	-0.64 ± 0.15	61.06 ± 0.24
	Re-sub.N3	-734.14 ± 55.20	4e-04	-0.51 ± 0.14	61.09 ± 0.24

Means ± SEM are shown, N = 70. *P*-values for modifications were determined by paired t-tests (μ = Real < Modification) on ΔG. *P*-values for randomizations were by one-sample t-tests (expected mean (μ) = 0) on Z(ΔG). %Pairs is the proportion of the coding sequence involved in base-pairing interactions. Artificial sequences generated by the first five protocols encode the same protein as the mouse sequence. A brief description of each protocol follows (see Results for details). 'Sh.4-fold': nucleotides at all 4-fold degenerate sites are shuffled. 'Sh.codon': for each amino acid, the synonymous codons are permuted. 'Re-sub.*K*': synonymous substitutions are reverted back to the rat-mouse common ancestor (rat-mouse common ancestor) state, followed by reallocation of the same number of synonymous point mutations. 'Re-sub.N3': like the previous protocol, except that the nucleotide replacement is also selected at random from the nucleotide distribution at third sites observed in the mouse sequence. 'Swap G4C4': all guanine bases at 4-fold sites are replaced by cytosine, and vice versa.
