# Supplementary material for: Evidence for selection on synonymous mutations affecting stability of mRNA secondary structure in mammals
Source: Genome Biol. 2005 Aug 16;6(9):R75. doi: 10.1186/gb-2005-6-9-r75 (PMC1242210; doi:10.1186/gb-2005-6-9-r75)
Supplement: Additional data file 2 — A table of the stability of mRNA secondary structures excluding the Gadd45a gene, where the rat-mouse common ancestor sequence differed slightly using the parsimony and maximum likelihood reconstructions. [file gb-2005-6-9-r75-S2.doc]

**Stability of mRNA secondary structures excluding the Gadd45a gene**

|  | Protocol | Mean ΔG | *P* | Mean Z(ΔG) | Mean %pairs |
| --- | --- | --- | --- | --- | --- |
| Real (mouse) |  | -742.73 ± 56.13 |  |  | 60.99 ± 0.28 |
| Modification | Swap G4C4 | -738.79 ± 55.68 | 0.0170 |  | 62.09 ± 0.33 |
| Randomization | Sh.4-fold | -730.24 ± 55.32 | 2e-15 | -1.44 0.14 | 60.80 ± 0.23 |
|  | Sh.codon | -733.06 ± 55.62 | 3e-10 | -1.06 0.14 | 60.58 ± 0.23 |
|  | Re-sub.*K* | -737.89 ± 55.76 | 2e-05 | -0.67 0.15 | 61.13 ± 0.25 |
|  | Re-sub.N3 | -738.75 ± 55.81 | 2e-04 | -0.54 0.14 | 61.16 ± 0.25 |

Means ±SEM are shown, N=69. *P*-values for modifications are determined by paired t-tests (μ=Real<Mod.) on ΔG. *P*-values for randomisations are by one-sample t-tests (expected mean (μ)=0) on Z(ΔG). %pairs is the proportion of the coding sequence involved in base-pairing interactions. Artificial sequences generated by the first five protocols encode the same protein as the mouse sequence (see Results).
