# Supplementary material for: Evidence for selection on synonymous mutations affecting stability of mRNA secondary structure in mammals
Source: Genome Biol. 2005 Aug 16;6(9):R75. doi: 10.1186/gb-2005-6-9-r75 (PMC1242210; doi:10.1186/gb-2005-6-9-r75)
Supplement: Additional data file 3 — A table of the stability of mRNA secondary structures for short genes (only considers mRNAs shorter than 2,000 nucleotides). [file gb-2005-6-9-r75-S3.doc]

Stability of mRNA secondary structures for short genes

|  | Protocol | Mean ΔG | *P* | Mean Z(ΔG) | Simulated *P* | *P* Mantell | Mean %pairs |
| --- | --- | --- | --- | --- | --- | --- | --- |
| Real |  | -410.90 ±27.36 |  |  |  |  | 60.57 ±0.37 |
| Mod. | Swap G4C4 | -408.03 ±26.72 | 0.12 |  | 0.1039 | 0.6938 | 61.92 ±0.48 |
| Rand. | Sh.4-fold | -402.04 ±26.55 | 2e-06 | -1.22 ±0.21 | 2e-07 | 0.9685 | 60.51 ±0.31 |
|  | Sh.codon | -403.42 ±26.67 | 3e-05 | -0.97 ±0.20 | 5e-05 | 0.6832 | 60.25 ±0.30 |
|  | Re-sub.*K* | -408.04 ±27.17 | 0.02 | -0.46 ±0.20 | 0.0126 | 0.8674 | 60.63 ±0.35 |
|  | Re-sub.N3 | -408.50 ±27.18 | 0.06 | -0.37 ±0.19 | 0.0342 | 0.8351 | 60.66 ±0.34 |

Means ±SEM are shown, N=36. *P*-values for modifications are determined by paired t-tests (μ=Real<Mod.) on ΔG. *P*-values for randomisations are by one-sample t-tests (expected mean (μ)=0) on Z(ΔG). %pairs is the proportion of the coding sequence involved in base-pairing interactions. Artificial sequences generated by the first five protocols encode the same protein as the mouse sequence (see Results). For each Mantell simulation, 10000 datasets of 36 randomly sampled genes were generated (see Materials and methods). Simulated *P* is the mean *P*-value from 10000 t-tests. *P* Mantell is the probability that one would observe a *P*-value less than that obtained from ignoring the long genes due to chance.
