# Supplementary material for: Evidence for selection on synonymous mutations affecting stability of mRNA secondary structure in mammals
Source: Genome Biol. 2005 Aug 16;6(9):R75. doi: 10.1186/gb-2005-6-9-r75 (PMC1242210; doi:10.1186/gb-2005-6-9-r75)
Supplement: Additional data file 7 — Miscellaneous correlations for short genes, including GC4 skew versus Z(ΔGSh.4-fold), GC12 skew versus GC3 skew (separately for base-paired and unpaired sites) and Z(ΔGRe-sub.N3) versus Ks at base-paired sites. [file gb-2005-6-9-r75-S7.doc]

Miscellaneous correlations for short genes

| X | Y | Correlation coefficient | *P* |
| --- | --- | --- | --- |
| GC4 skew | Z(ΔG^Sh.4-fold^) | ρ=0.22 | 0.20 |
| GC12 skew base-paired | GC3 skew base-paired | *R*=−0.72 | 8e-07 |
| GC12 skew unpaired | GC3 skew unpaired | *R*=0.67 | 7e-06 |
| Z(ΔG^Re-sub.N3^) | *K*_s_ base-paired | ρ=−0.40 | 0.02 |

N=36, except for Z(ΔG^Re-sub.N3^) versus *K*_s_ base-paired (N=35). *R*=Pearson correlation coefficient, ρ=Spearman rank correlation coefficient.
