# Supplementary material for: Evidence for selection on synonymous mutations affecting stability of mRNA secondary structure in mammals
Source: Genome Biol. 2005 Aug 16;6(9):R75. doi: 10.1186/gb-2005-6-9-r75 (PMC1242210; doi:10.1186/gb-2005-6-9-r75)
Supplement: Additional data file 8 — A table of correlations between GC skew at first/second sites versus skew at third sites, provided for a series of thresholds where the sites analyzed must have a minimum probability of base-pairing. [file gb-2005-6-9-r75-S8.doc]

Relationships between GC12 skew and GC3 skew for a series of minimum base-pairing probabilities

| Minimum *P* | *P*(*i*,*j*) |  |  | Total *P*(*i*) |  |  |
| --- | --- | --- | --- | --- | --- | --- |
|  | N | *R* | *P* | N | *R* | *P* |
| 0 | 70 | -0.65 | 1e-09 | 70 | -0.65 | 1e-09 |
| 0.1 | 70 | -0.46 | 5e-05 | 70 | -0.60 | 5e-08 |
| 0.2 | 70 | -0.49 | 1e-05 | 70 | -0.56 | 4e-07 |
| 0.3 | 70 | -0.55 | 8e-07 | 70 | -0.54 | 1e-06 |
| 0.4 | 70 | -0.57 | 3e-07 | 70 | -0.52 | 5e-06 |
| 0.5 | 70 | -0.55 | 7e-07 | 70 | -0.40 | 0.0005 |
| 0.6 | 69 | -0.60 | 6e-08 | 69 | -0.44 | 0.0001 |
| 0.7 | 68 | -0.52 | 5e-06 | 68 | -0.38 | 0.0015 |
| 0.8 | 67 | -0.33 | 0.0061 | 67 | -0.32 | 0.0073 |
| 0.9 | 64 | -0.30 | 0.0171 | 66 | -0.14 | 0.2686 |

GC skew is the bias in cytosine or guanine usage (G-C/(G+C)). *P*(*i*,*j*) is the probability of base-pairing between sites *i* and *j* in the predicted optimal secondary structure. Total *P*(*i*) is the total probability for site *i* being paired with any other site. For each minimum *P­*, a given site is only considered when calculating GC skew if its probability of pairing is greater than or equal to the minimum *P*-value.
