# Supplementary material for: Evidence for selection on synonymous mutations affecting stability of mRNA secondary structure in mammals
Source: Genome Biol. 2005 Aug 16;6(9):R75. doi: 10.1186/gb-2005-6-9-r75 (PMC1242210; doi:10.1186/gb-2005-6-9-r75)
Supplement: Additional data file 10 — A table of correlations for short genes, between the proportion of base-paired sites and non-synonymous or synonymous substitution rates within the coding sequence, base-paired sites and unpaired sites. [file gb-2005-6-9-r75-S10.doc]

Relationships between the proportion of base-paired sites in mouse coding sequence and rates of evolution for short genes

| Site | *K*_a_ |  |  | *K*_s_ |  |  |
| --- | --- | --- | --- | --- | --- | --- |
|  | Mean ± SEM | ρ | *P* | Mean ± SEM | ρ | *P* |
| CDS | 0.0105 ±0.0019 | 0.14 | 0.42 | 0.0683 ±0.0048 | 0.41 | 0.01 |
| Base-paired | 0.0097 ±0.0019 | 0.22 | 0.21 | 0.0562 ± 0.0052 | 0.57 | <0.01 |
| Unpaired | 0.0127 ±0.0030 | –0.08 | 0.67 | 0.0895 ± 0.0124 | –0.09 | 0.60 |

N=35. *K*_a_=non-synonymous substitution rate, *K*_s_= synonymous substitution rate. ρ=Spearman rank correlation coefficient.
